# Protein-protein interaction based on pairwise similarity

**DOI:** 10.1186/1471-2105-10-150

**Published:** 2009-05-17

**Authors:** Nazar Zaki, Sanja Lazarova-Molnar, Wassim El-Hajj, Piers Campbell

**Affiliations:** 1 Bioinformatics Laboratory, Department of Computer Science, College of Information Technology, UAE University, Al Ain 17551, UAE; 2 Department of Information Security, College of Information Technology, UAE University, Al Ain 17551, UAE; 3 Department of Information System, College of Information Technology, UAE University, Al Ain 17551, UAE

## Abstract

**Background:**

Protein-protein interaction (PPI) is essential to most biological processes. Abnormal interactions may have implications in a number of neurological syndromes. Given that the association and dissociation of protein molecules is crucial, computational tools capable of effectively identifying PPI are desirable. In this paper, we propose a simple yet effective method to detect PPI based on pairwise similarity and using only the primary structure of the protein. The PPI based on Pairwise Similarity (PPI-PS) method consists of a representation of each protein sequence by a vector of pairwise similarities against large subsequences of amino acids created by a shifting window which passes over concatenated protein training sequences. Each coordinate of this vector is typically the E-value of the Smith-Waterman score. These vectors are then used to compute the kernel matrix which will be exploited in conjunction with support vector machines.

**Results:**

To assess the ability of the proposed method to recognize the difference between "*interacted*" and "*non-interacted*" proteins pairs, we applied it on different datasets from the available yeast *saccharomyces cerevisiae *protein interaction. The proposed method achieved reasonable improvement over the existing state-of-the-art methods for PPI prediction.

**Conclusion:**

Pairwise similarity score provides a relevant measure of similarity between protein sequences. This similarity incorporates biological knowledge about proteins and it is extremely powerful when combined with support vector machine to predict PPI.

## Background

Protein-protein interaction is intrinsic to most cellular processes and can aid significantly in identifying the function of newly discovered proteins and in understanding the molecular networks they participate in [1]. Therefore, one of the major remaining goals in functional genomics is to determine protein interaction networks for the whole organism. To solve this problem, a vast set of impressive experimental techniques has been developed to predict the physical interactions which could lead to the identification of the functional relationships between proteins. These techniques include; yeast two-hybrid-based methods [2]; mass spectrometry [3]; Tandem Affinity Purification [4]; protein chips [5]; and hybrid approaches [6]. These techniques have assisted in identifying hundreds of potential interacting proteins in several species such as Yeast, Drosophila, and Helicobacter-pylori [7]. They are however, both very expensive and significantly time consuming and to date the PPI pairs obtained cover only a fraction of the complete PPI network.

The technical limitations associated with the mentioned biochemical approaches have resulted in a growing need for development of computational tools that are capable of identifying PPIs [8]. Hence, computational approaches remain essential both to assist in design and validation of experimental studies and for the prediction of interaction partners and detailed structures of protein complexes [9]. As a result, a number of computational methods have been developed. A number of the earlier computational methods were based on genomic information, such as similarity of expression profiles [10], phylogenetic profiles [11,12] or phylogenetic trees [13] and adjacency of genes [14]. However, the main limitations of such methods are that they can be applied only to completely sequenced gene and can be used only with essential proteins that are common to most organisms [7].

Most of the recent computational methods employ domain knowledge to predict the PPI. The motivation behind this employment is that molecular interactions are typically mediated by a great variety of interacting domains [15]. Sprinzak *et al*. [1] developed the Association Method (AM) which defines a simple measure of interaction probability between two domains as the fraction of interacting protein pairs among all protein pairs containing the domain pairs. The limitation of this method lies in the possibility to assign high association scores to domain pairs with low frequency. Deng *et al*. [16] developed the Maximum Likelihood Estimation (MLE) method which is based on the assumption that two proteins interact if at least one pair of domains of the two proteins interact. Huang *et al*. [17] introduced the Maximum Specificity Set Cover (MSSC). Huang started by selecting high quality protein interactions based on a clustering measure and then used MSSC to assign probabilities to domain pairs. As most of the existing domain-based methods consider only single-domain pairs and assume independence between domain-domain interactions, Xue-Wen *et al*. [18] introduced a domain-based random forest of decision trees to infer protein interactions. This method is capable of exploring all possible domain interactions and making predictions based on all the protein domains.

A recent tool termed PIPE (Protein-Protein Interaction Prediction Engine) was developed by Sylvain *et al*. [8]. PIPE is based on the assumption that some of the interactions between proteins are mediated by a finite number of short polypeptide sequences. These sequences are typically shorter than the classical domains, and are used repeatedly in different proteins and contexts within the cell. Once the interaction database is large enough to sample these sequences, it should be possible to accurately predict such PPIs. PIPE uses the primary structure of proteins together with the available protein interaction data to predict the potential interaction between any target pair of *saccharomyces cerevisiae *proteins.

Most of the methods discussed have common limitations:

• They are based on previously identified domains, and the identification of domain is a long and computationally expensive process.

• They all focus on domain structure and none considers the complete sequence information. We understand that protein domains are highly informative for predicting PPI as they reflect the potential structural relationships between proteins. However, other sequence parts (not carrying any domain knowledge) may also contribute significantly to the information by showing differences between proteins.

• They are not universal because their accuracy and reliability is dependant on the domain information or interaction marks of the protein partners.

• They often have limited abilities to detect novel interactions and to differentiate them from false positives. A high rate of false negatives is another disadvantage associated with most of these methods.

In this paper, we introduce a simple yet effective method to predict PPI based on pairwise similarity and using only protein primary structure. Two proteins may interact by the means of the scores similarities they produce against subsequences of amino acids created by a large shifting window which passes over concatenated protein training sequences. This work is motivated by the observation that the pairwise score, which measures the similarity between two protein sequences by a local gapped alignment, provides a relevant measure of similarity between protein sequences. This similarity incorporates biological knowledge about protein evolutionary structural relationships [19].

## Results

In our first experimental work, we tested the performance of our method on randomly selected 15 protein sequences from the yeast protein interaction. The datasets are prepared as listed in Table 1. The mean, standard deviation and confidence level (95%) of the length of the training and testing datasets are listed in Table 2.

**Table 1:** Randomly selected training and testing protein datasets

**Training Dataset**		**Testing Dataset**	
YAR003W-YBR175W	interact	YCR077C-YDL160C	interact
YBR126C-YML100W	interact	YPR072W-YIL038C	interact
YNR006W-YOR025W	non-interact	YNL137C-YOR025W	non-interact
YMR203W-YNL029C	non-interact	YMR261C-YOR321W	non-interact

**Table 2:** Mean, standard deviation and confidence level of the length of the selected 15 proteins

	**Mean**	**Standard Deviation**	**Confidence level (95%)**
Training Dataset	539	243.81	203.83
Testing Dataset	679.75	213.67	178.64

The goal of this experiment is to confirm that two sequences may interact if they are similar, but one must also be careful that the training and testing sets are sequence independent. Therefore, for each sequence (*s*_*ts*_) in the (*m*) testing set (*ts*), we calculate the similarity sores (*sc*_*tstr*_) against each of the other (*n*) sequences in the training set (*tr*). The default alignment parameters are used; gap opening penalty and extension penalties of 11 and 1, respectively, and the BLOSUM 62 matrix. The similarity averages (*μ*_*s*_) are calculated and finally the average (*μ*_*SIM*_) and standard deviation (*σ*_*SIM*_) of all averages are recorded. The process is illustrated as follows:

(1)

We understand that similarity score is meaningful information when comparing protein sequences as it is derived from accumulated knowledge of both protein structure and function. However, similarity score is difficult to interpret as it is not normalized on length. Therefore, we calculated the identity scores averages (*μ*_*id*_) and then the average (*μ*_*ID*_) and standard deviation (*σ*_*ID*_) of all averages are recorded. For each sequence (*s*_*ts*_) in the testing set we align it against each sequence in the training set, count the number of positions that have identical amino acids and then divide by the total length of the alignment. The process is illustrated as follows:

(2)

The maximum identity score (*si*_max_) for each sequence in the testing set against each sequence in the training set is identified and the average (*μ*_*MAX*_) and standard deviation (*σ*_*MAX*_) of the (*si*_max_) are then reported. The process is illustrated as follow:

(3)

The final averages and standard deviations calculated from (1), (2) and (3) are summarized in Table 3.

**Table 3:** Similarity and identity averages and standard deviations calculated based on the selected 15 proteins

**Similarity**		**Identity**		**Maximum Identity**	
*μ*_*SIM*_	*σ*_*SIM*_	*μ*_*ID*_	*σ*_*ID*_	*μ*_*MAX*_	*σ*_*MAX*_
48.35	3.79	29.59	2.72	51.97	15.09

This information shows that on average proteins in the testing set have realistic similar homologies in the training set.

The feature extraction step starts by creating a long string of amino acids by concatenating all of the 8 protein sequences available in the training dataset. By choosing a large window of size 1500, we were able to generate 3 subsequences of lengths 1500, 1500 and 312, respectively (the total length *l *in this case is 3312 amino acids). All protein sequences in the training and testing datasets were scored against the 3 generated subsequences using Smith-Waterman (SW) algorithm as implemented in Fasta [20]. The SW [21] has undergone two decades of empirical optimization in the field of bioinformatics and thus, considerable prior knowledge is implicitly incorporated into the pairwise sequence similarity scores and hence into the PPI-PS vector representation. For instance, if we have a protein sequence *s *then the corresponding score will be  where *m*-1 is the total number of proteins and  is the E-value of the SW score between sequence *s *and the *i*^*th *^subsequence. In this case, the default parameters are used; gap opening penalty and extension penalties of 13 and 3, respectively, and the BLOSUM 62 matrix. Based on prior biological knowledge about the interaction information between proteins, the feature vectors of two "*interacted*" proteins *s*_0 _and *s*_1 _are concatenated and added to the positive set, and the "*non-interacting*" proteins are also concatenated and added to the negative set for both training and testing datasets.

Following the preparation of the training and testing sets, we employed Gist SVM to discriminate between the "*interacted*" and "*non-interacted*" proteins in the testing dataset. The Gist SVM software is implemented by Noble *et al*. and it is available at . In all experiments, Gaussian Radial Basis Function kernel (RBF kernel) was used, the RBF kernel allows pockets of data to be classified which is more powerful approach than simply using a linear dot product [22,23]. The function has the form , where *x*, *x*_*i *_∈ *X *and *γ *> 0. In all of the experimental work, the scaling parameter *γ *was set to 0.001.

**Figure 1:**
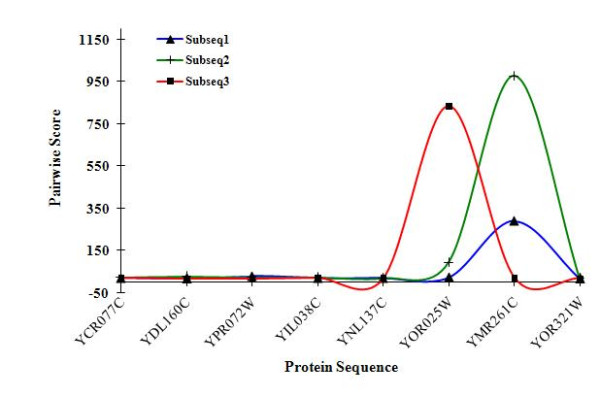
**Similarity score of each protein sequence in the testing dataset against the three generated subsequences**.

The accuracies of our predictions are measured by specificity (SP), sensitivity (SN) and the receiver operating characteristic (ROC). The specificity is defined as the ratio of the number of matched interactions between the predicted set, and the observed testing set, over the total number of predicted interactions. The sensitivity is defined as the ratio of the number of matched interactions to the total number of observed interactions in the testing set [17]. The ROC is the fraction of true positives (TPR = true positive rate) vs. the fraction of false positives (FPR = false positive rate). In this particularly straightforward experimental work, we were able to achieve overall accuracy of 100%. However it's pertinent to provide more analysis of the algorithm's performance and results. In Figure 1, we summarize the similarity score of each protein sequence in the testing dataset against the three generated subsequences.

Figure 1 clearly shows that the four proteins (YCR077C-YDL160C and YPR072W-YIL038C) that belong to the positive set obtained low scores against the 3 subsequences. Moreover, the remaining 4 proteins (YNL137C-YOR025W and YMR261C-YOR321W) scored relatively high.

In our second experimental work, we assess the recognition ability of our method in classifying between 100 interacted protein pairs (157 proteins) and 100 non-interacted protein pairs (77 proteins). The dataset used was randomly selected by Sylvain *et. al *[8] and used to evaluate PIPE's accuracy. It was generated from the yeast protein interaction literature for which at least three different lines of experimental evidence supported the interaction.

The mean, standard deviation and confidence level (95%) of the length of the 157 positive and 77 negative protein sequences are listed in Table 4. The averages and standard deviations calculated from (1), (2) and (3) are summarized in Table 5.

**Table 4:** Mean, standard deviation and confidence level of the length of the 157 positive and 77 negative protein sequences

	**Mean**	**Standard Deviation**	**Confidence level (95%)**
Positive Examples	567.7	374.7	59.08
Negative Examples	510.27	314.15	71.30

**Table 5:** Similarity and identity averages and standard deviations calculated based on the 157 positive and 77 negative protein sequences

**Similarity**		**Identity**		**Maximum Identity**	
*μ*_*SIM*_	*σ*_*SIM*_	*μ*_*ID*_	*σ*_*ID*_	*μ*_*MAX*_	*σ*_*MAX*_
44.97	2.26	29.21	2.41	65.57	10.93

These results show that on average proteins in the testing set have high similar homologies to those in the training set.

We created a long string of amino acids by concatenating all of the 234 protein sequences (157+77 sequences). Various window sizes are used to generate various amino acid subsequences. All of the 234 protein sequences were scored against the generated subsequences. The experimental set up used was similar to that mentioned in the previous experiment, with the exception that hold-one-out cross-validation was employed to measure the accuracy.

In Table 6, we record the ROC, SN, SP and the overall accuracy based on various window sizes. The results show that window size equal to 5000 produced better ROC, SN, SP and overall accuracy results. The proposed method shows an improvement over PIPE. PIPE produced a SN of 0.61 for detecting yeast protein interaction with 0.89 SP and an overall accuracy of 0.75.

**Table 6:** ROC, SN, SP and overall accuracy recorded from testing PPI-PS on 100 interacting protein pairs and 100 non-interacting protein pairs based on several window size values.

**Window size**	**ROC**	**SN**	**SP**	**Accuracy**
20000	0.9591	0.9	0.9	0.9
19000	0.9751	0.94	0.86	0.9
18000	0.996	1	0.96	0.98
17000	0.976	0.95	0.92	0.935
16000	0.974	0.88	0.91	0.895
15000	0.996	1	0.96	0.98
14000	0.979	0.91	0.97	0.94
13000	0.9918	1	0.94	0.97
12000	0.98804	0.93	0.97	0.95
11000	0.9885	0.98	0.96	0.97
10000	0.9985	1	0.95	0.975
9000	0.9979	1	0.95	0.975
8000	0.989	0.98	0.98	0.98
7000	0.9964	1	0.93	0.965
6000	0.9984	1	0.95	0.975
**5000**	**0.9991**	**1**	**0.98**	**0.99**
4000	0.9941	0.98	0.96	0.97
3000	0.9962	1	0.95	0.975
2000	0.9927	0.97	0.94	0.955
1000	0.9864	0.96	0.87	0.915
500	0.973	0.96	0.78	0.87

To insure the effect of the leave-one-out evaluation, further investigation was conducted by creating a long string of amino acids by concatenating all of the 234 protein sequences except for the two sequences which are to be classified. The results in this case show no statistical difference in accuracy (results not shown). This is most probably due to the fact that we eliminate only one feature vector.

In addition to its superior accuracy, PPI-PS has two further advantages when compared to PIPE. Firstly, the PIPE method is computationally intensive and the evaluation of PIPE's performance over the same dataset took approximately 1,000 hours of computation time compared to only a few minutes using PPI-PS [8].

Secondly, as is mentioned by the PIPE's authors, their method is expected to be weak if it is used for detecting novel interactions among genome wide large-scale data sets. This is not true for PPI-PS as can be seen in the subsequent sections of this paper which describe a large scale data test.

For the third experiment we furthermore split the 100 interacted protein pairs into two sets A (50 pairs) and B (50 pairs). We also split the 100 non-interacted protein pairs into two sets C (50 pairs) and D (50 pairs). We then combined A with C to create a training dataset and B with D to create a testing dataset. A set up similar to that describe for experiment 1 was employed. In Table 7 we show ROC, SN, SP and overall accuracy calculated using several window sizes (*n*). Values of *n *= 12000 and 13000 yielded more accurate results.

**Table 7:** ROC, SN, SP and overall accuracy recorded from testing PPI-PS on a dataset of 50 interacting protein pairs and 50 non-interacting protein pairs based on several window size values.

**Window size**	**ROC**	**SN**	**SP**	**Accuracy**
20000	0.8648	0.48	0.86	0.67
19000	0.87772	1	0.78	0.89
18000	0.8432	0.96	0.78	0.87
17000	0.8336	0.88	0.76	0.82
16000	0.8176	0.78	0.76	0.77
15000	0.8612	0.82	0.76	0.79
14000	0.854	1	0.74	0.87
**13000**	**0.8784**	**0.98**	**0.82**	**0.9**
**12000**	**0.8632**	**1**	**0.8**	**0.9**
11000	0.842	1	0.72	0.86
10000	0.8556	0.8	0.8	0.8
9000	0.8628	0.94	0.78	0.86
8000	0.8724	0.96	0.72	0.84
7000	0.8732	0.98	0.76	0.87
6000	0.8812	1	0.74	0.87
5000	0.8792	0.96	0.74	0.85
4000	0.8532	1	0.72	0.86
3000	0.8876	1	0.74	0.87
2000	0.8488	1	0.62	0.81
1000	0.8608	1	0.58	0.79
500	0.8544	1	0.46	0.73

We understand that embedding pairs of protein sequences in a vector space could be order dependent [24] and therefore, we ran additional experimental work to check the order dependency for sequence pairs. We classified the testing pairs with the order of the sequence reversed. Instead of classifying the concatenation of *s*_1 _to *s*_2_, we classified *s*_2 _to *s*_1_. We then reported the average accuracy and compared it to the original order average. Table 8 summarizes this comparison and the results show no statistical effect on the accuracy.

**Table 8:** Comparing the classification accuracy of the 200 protein pairs based on reversed sequence order.

**Window size**	**ROC**	**SN**	**SP**	**Accuracy**
Original Order	0.86	0.93	0.735	0.833
Reverse Order	0.853	0.93	0.73	0.83

In the fourth experimental work, we assess the recognition ability of our method on the dataset created by Xue-Wen *et al*. [18]. Xue-Wen initially obtained 15,409 interacting protein pairs in the yeast organism from DIP, 5719 pairs from Deng *et al*. [16] and 2238 pairs from Schwikowski *et al*. [25]. The datasets were then combined by removing the overlapping interaction pairs and excluding the pairs for which at least one of the proteins had no domain information. Finally, 9834 protein interaction pairs remained among 3713 proteins, and they were separated evenly (4917 pairs each) into training and testing datasets. As non-interacting protein data are unavailable, the negative samples are randomly generated. A protein pair is considered to be a negative sample if the pair does not exist in the interaction set. A total of 8000 negative samples were generated and also separated into two halves. Both final training and testing datasets contain 8917 samples, 4917 positive and 4000 negative samples.

The mean, standard deviation and confidence level (95%) of the length of the 8917 training examples and the 8917 testing examples are listed in Table 9. The averages and standard deviations calculated from (1), (2) and (3) are summarized in Table 10.

**Table 9:** Mean, standard deviation and confidence level of the length of the 8917 training examples and the 8917 testing examples

	**Mean**	**Standard Deviation**	**Confidence level (95%)**
Training Examples	548.31	398.29	12.10
Testing Examples	547.48	398.27	12.14

**Table 10:** Similarity and identity averages and standard deviations calculated based on the 8917 training examples and the 8917 testing examples

**Similarity**		**Identity**		**Maximum Identity**	
*μ*_*SIM*_	*σ*_*SIM*_	*μ*_*ID*_	*σ*_*ID*_	*μ*_*MAX*_	*σ*_*MAX*_
19.16	9.91	29.97	1.96	81.59	6.71

This information shows that on average proteins in the testing set have high similar homologies in the training set. In Table 11 we show ROC, SN, SP and overall accuracy calculated using various window sizes (*n*). Values of *n *= 5000 produced more accurate results.

**Table 11:** ROC, SN, SP and overall accuracy recorded from testing PPI-PS on a testing dataset of 4917 interacting protein pairs and 4000 non-interacting protein pairs based on several window size values.

**Window size**	**ROC**	**SN**	**SP**	**Accuracy**
20000	0.8407	0.7914	0.7357	0.7664
15000	0.84	0.793	0.736	0.767
10000	0.845	0.795	0.745	0.77
**1000**	**0.8534**	**0.807**	**0.744**	**0.7789**
500	0.7858	0.7	0.721	0.7098

We further compared the classification accuracy averages based on the original and reverse protein order as discussed in the third experiment. Table 12 summarizes the comparison. The results show no significant statistical effect on the accuracy.

**Table 12:** Comparing the classification accuracy of the 8917 protein pairs based on reversed sequence order.

**Window size**	**ROC**	**SN**	**SP**	**Accuracy**
Original Order	0.833	0.77	0.73	0.75
Reverse Order	0.80	0.71	0.726	0.72

For comparative purposes, we tested two further state-of-the-art sequence based methods, maximum likelihood estimation (MLE) developed by Deng *et al*. [16] and domain-based random forest of decision trees, developed by Xue-Wen *et al*. [18].

**Figure 2:**
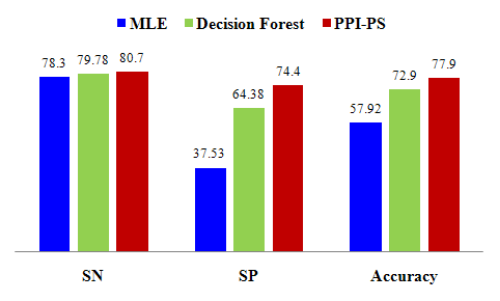
**Comparing PPI-PS performance with MLE and Domain-based random forest of decision trees methods**.

The results of the primary experiment with a window size *n *= 1000 are summarized in Figure 2. The figure also shows a performance comparison between PPI-PS and the other two state-of-the-art sequence based methods; MLE and Domain-based random forest of decision trees. Higher SN, SP and overall accuracy correspond to more accurate PPI detection performance. Selecting any of these performance measures, it is clear that the PPI-PS method performs better than the other methods.

## Discussion

The method presented in this paper is based on the assumption that two proteins may interact if their pairwise scores against large subsequences of amino acids created by shifting a window over concatenated protein training sequences are similar. It is commonly understood that, this assumption excludes the applicability of interactions of proteins which are not similar or evolutionary related to each other. However, the main contribution of this paper is to show that pairwise sequence comparison can be extremely powerful when combined with Support Vector Machines (SVM).

Moreover, we are motivated by the fact that SW alignment score provides a relevant measure of similarity between proteins. Therefore protein sequence similarity typically implies homology, which in turn may imply structural and functional similarity [26]. The experimental results have shown that the PPI-PS method applied to different datasets from the yeast saccharomyces cerevisiae protein interaction literature can predict PPIs with higher specificity and sensitivity than PIPE, MLE and decision forest methods.

The detailed properties of the protein datasets used in this research work are presented and analysed. The average maximum sequence identity between testing and training datasets is considered high which could confirm that two sequences may interact if they are similar. In addition, this could also make the prediction task easier as in this case many of the test sequences have homologs in the training dataset. Ideally, one would create a dataset in which there is low sequence similarity between members in order to prove generalizability, however, in this study we used previously established benchmark datasets in order to make our results directly comparable.

The remarkable accuracy of our method follows from the combination of two widely used and significantly powerful algorithms. Firstly, the SVM algorithm is based on a sound mathematical framework and much of its power is derived from its criterion for selecting a separating hyperplane that maintains a maximum margin from any point in the training set [27]. Secondly, SW scores have been developed to quantify the similarity of biological sequences. Their parameters have been optimized over the past two decades to provide relevant measures of similarity between sequences and they now represent core tools in computational biology [19].

One significant characteristic of any protein-protein interaction prediction algorithm is the computation efficiency. In order to gauge the computational cost of the proposed approach, PPI-PS includes SVM optimization, whose complexity is roughly *O*(*n*^2^), where *n *in this case is the number of training set examples [26]. The feature sensitivity measure step of PPI-PS method involves computing *n*^2 ^pairwise scores. Using SW itself is computed by dynamic programming and each computation is *O*(*m*^2^), where *m *is the length of the longest sequence in the training set.

The method presented here is encouraged by the success of our earlier method, SubSS [28] which was used to distinguish between high confidence protein interaction pairs and low confidence or unknown protein interaction pairs. Despite the fact that SubSS has shown considerable accuracy improvement over the existing techniques, SubSS has three main limitations: (a) the size of the sliding window used and analyzed was very small. Small size subsequence of amino acid is likely to appear in many protein sequences makes it difficult to judge whether the two proteins are actually interacting or not. (b) Sliding a small window size yields more subsequences which significantly increase the computational time. Since each SW computation cost is *O*(*m*^2^), the total cost to compare *α *protein sequences to *β *subsequences is *O*(*m*^2^·*α*·*β*). Small window size will increase the value of *β*. (c) The results produced by SubSS are not stable since the method is designed to randomly select several negative examples from the low confidence protein interacting pairs every time the method runs. In this work only large values of *n *were used and analyzed. The method is intensively tested and validated by comparing it with highly respected existing methods.

It is important to mention that the idea of representing protein sequence via its similarity to a collection of other sequences is not novel. Liao *et al*. [26] and Zaki *et al*. [29] have conducted similar work in their algorithm to detect protein remote homology.

## Conclusion

Protein-protein interaction has proven to be a valuable biological knowledge and starting point for understanding how the cell internally works. In this study we propose a method for PPI prediction using only the primary structure information of protein sequence. The method was developed based on a combination of pairwise similarity and support vector machine. It is shown that pairwise similarity score provides relevant measure of similarity between protein sequences. This similarity incorporates biological knowledge about proteins and its extremely powerful when combined with support vector machine to predict PPI.

Finally, the success of the PPI-PS method at predicting PPI encouraged us to plan future investigations such as optimizing the subsequence size and applying the method on gold standard positive (GSP) and negative (GSN) interaction sets recently created by Ramazan *et al*. [30]. We understand that similarity is not the only evidence of protein interaction; however, researchers have not intensively tested how much evidence similarity could provide. A combination of knowledge about gene ontology (GO), inter-domain linker region and interacting sites may significantly improve the prediction accuracy.

## Methods

The PPI based on Pairwise Similarity (PPI-PS) method consists of two major steps:

◦ Feature extraction step: representing each protein sequence by a vector of pairwise similarities against subsequences of amino acids.

◦ Classification: taking as a kernel the dot product between these vector representations to be used in conjunction with SVM.

In the proceeding sections, we describe both steps.

### Protein feature extraction

In the feature extraction step, we represent a protein sequence by a fixed-length of feature vectors. Each coordinate of this feature vector is typically the E-value of the SW score created by shifting a window over the protein training sequences. This step is formulated as follows:

We begin by providing the symbols and sets used for describing the algorithm:

◦ *S *– set of protein sequences of interest

◦ S* = {*s*_0_, *s*_1_, ..., *s*_*m*-1_} – enumerated set of protein sequences in the database, S* ⊆ *S*

◦ *B *= {*T*, *F*} – is the Boolean domain.

Further, we define the following functions and operators:

• *Interact*: *S *× *S *× *P*(*S*) → *B*, where *P*(*S*) is the power set of *S*.

◦ *Interact*(*s*_1_, *s*_2_, S*) – checks whether two protein sequences *s*_1 _and *s*_2 _interact, if true returns *T*, else returns *F *and if non-existent returns null.

• *concatenate*: *S *× *S *→ *S*

◦ *concatenate*(*s*_1_, *s*_2_) – merges two sequences *s*_1 _and *s*_2 _in the order they are specified and returns the resulting sequence

◦ E.g. *concatenate*(*acd*, *efg*) = *acdefg*

• *concatenate*_*set*_: *P*(*S*) → *S*

◦ *concatenate*_*set *_(*A*) – merges all sequences in A and returns a long string of amino acid

◦ E.g. *A *= {*acd*, *efg*, *am*}, *concatenate*(*A*) = *acdefgam*

• *length*: *S *→ *N*

◦ *length*(*s*) – returns the length of the sequence *s*

• *addseq*(*e*, *A*) – adds the element *e *to the set *A*

• *subseq*: *S *× *N *× *N *→ *S*

◦ *subseq*(*s*, *d*, *n*) – returns subsequence of *s *that starts at position *d *and has a window size *n*

◦ E.g. *subseq*(*abdefg*, 2,3) = *def*

• *merge*(*a*, *b*) – merges two row-vectors a and b, and returns the resulting row-vector

◦ E.g. *merge*([1,3],[4,5]) = [2,3,4,5]

**Figure 3:**
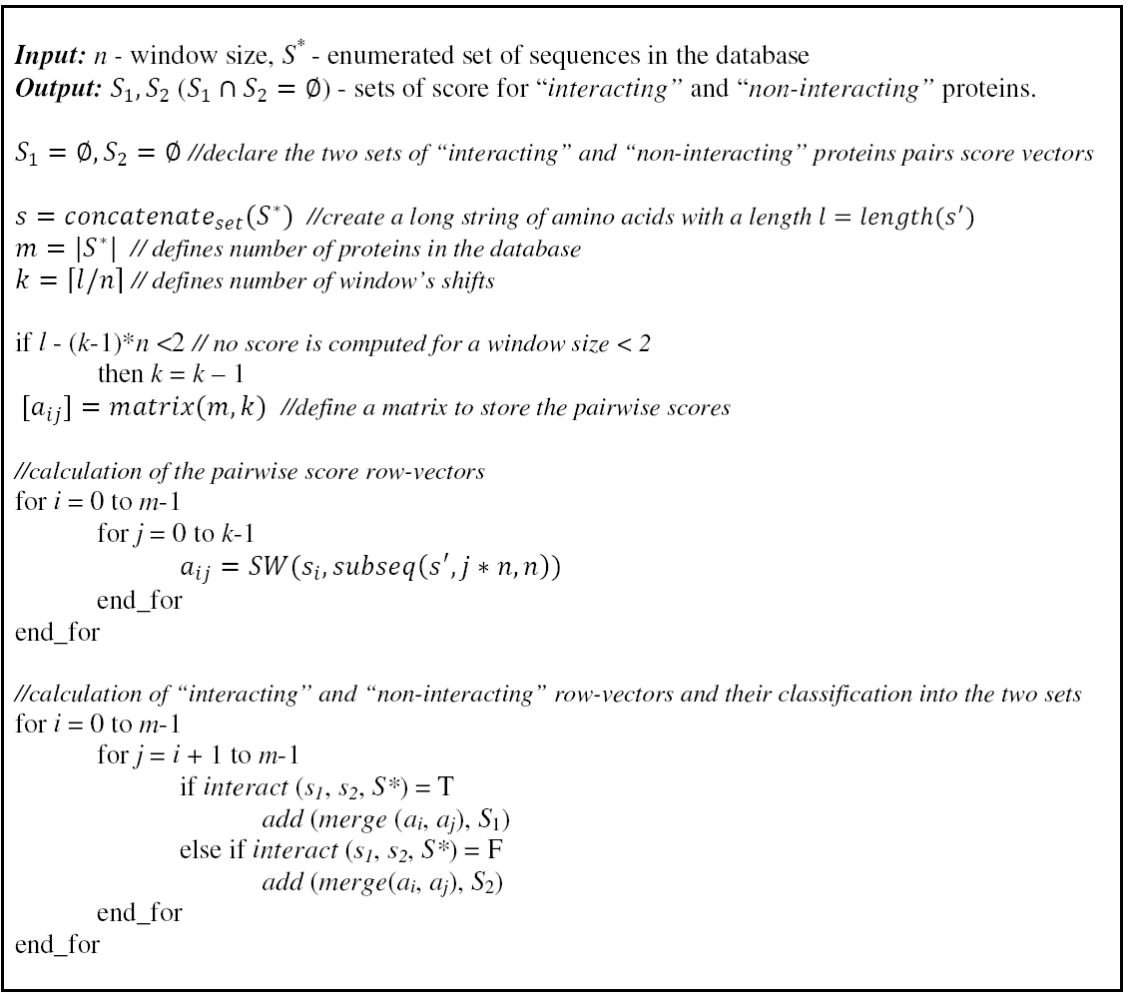
**Illustration of the feature extraction algorithm**.

The algorithm for feature extraction is illustrated in Figure 3.

### Smith-Waterman score

The Smith-Waterman score *SW*(*s*_0_, *s*_1_) between protein sequences *s*_0 _and the subsequence s_1 _is the score of the best local alignment with gaps between the two protein sequences, computed by the SW dynamic programming algorithm [21].

Following Saigo *et al*. [19], let us denote by *π *a possible local alignment between protein sequences *s*_0 _and the subsequence *s*_1_, defined by a number *n *of aligned residues, and by the indices 1 ≤ *i*_1 _< ... <*i*_*n *_≤ |*s*_0_| and 1 ≤ *j*_1 _< ... <*j*_*n *_≤ |*s*_1_| of the aligned residues in *s*_0 _and *s*_1 _respectively. Let us also denote by ∏(*s*_0_, *s*_1_) the set of all possible local alignments between *s*_0 _and *s*_1_, and by *p*(*s*_0_, *s*_1_, *π*) the score of the local alignment *π *∈ ∏(*s*_0_, *s*_1_) between *s*_0 _and *s*_1_, the Smith-Waterman score *SW*(s_0_, s_1_) between *s*_0 _and *s*_1 _can be written as .

The process is illustrated in the following example:

Let us assume that the set of enumerated protein sequences *s*_0_, *s*_1_, *s*_2_, and *s*_3 _in the database is S* = {*admn,qghk,il,ged*}. The number of elements in S* is *m *= 4. We also assume that we have prior knowledge about the interaction information between these proteins:



We choose a window size *n *= 4. We then concatenate all sequences in S* to obtain *s' *= *admngqhki*lg *ed *with length *l *= 13. Next, we compute the pairwise score between each protein sequence in S* and the substrings created by shifting a window of a size *n *along *S*. It's common in bioinformatics to slide a window by a single position however this will generate more subsequences than simply shifting the window by its size. For instance, sliding a window of size 4 over *s' *yields 10 subsequences, however shifting it by its size yields only 3 subsequences. The two notions have been tested and the results suggested no significant differences in accuracy (results not shown). Using a shifting window over the concatenated sequences of the training set may lead to generating a subsequence comprises of the end of one sequence and the beginning of the next sequence. This, however, is not a problem since all protein sequence of interest score against the same subsequence.

The results are then stored in a matrix of size *m *× *k*. In this case *k *= 3, because the size of the last window shift of *s' *is 1, i.e. score cannot be computed for a sequence of size less than 2 amino acids. This implies that, the resulting matrix will have a size of 4 × 3. After that, the score vectors of the "*interacting*" proteins are merged and added to the set *S*_1_, and the ones of the "*non-interacting*" proteins are added to the set *S*_2_.

**Figure 4:**
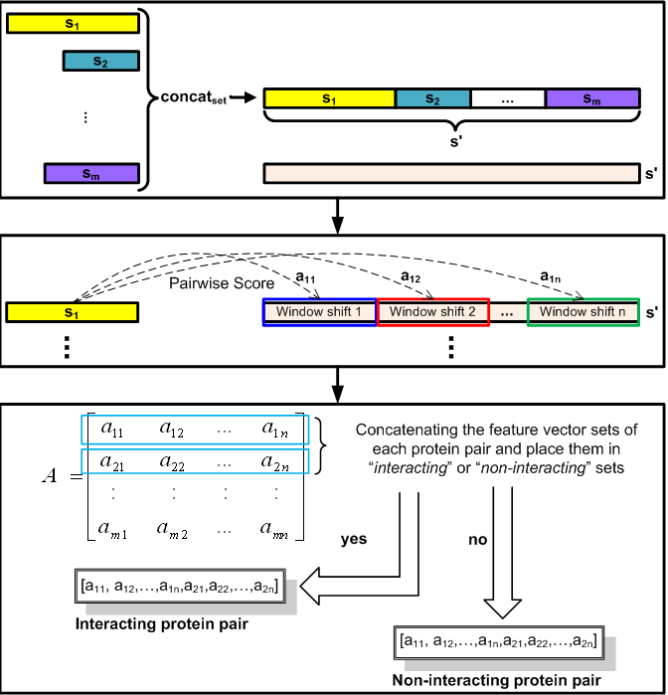
**Overview of the feature extraction step**.

We believe that the feature extraction is particularly significant step in our method to predict PPI. More meaningful features yield better generalization performance [27]. The feature extraction process is further illustrated in Figure 4.

### Classification Step

The problem is basically formulated as a two-class classification problem: both training and testing sets contain protein pairs belong to either "*interacted*" or "*non-interacted*". This representation is combined with SVM to classify between the two sets. The SVM algorithm addresses the general problem of learning to discriminate between positive and negative examples of a given class of *n*-dimensional vectors. In order to discriminate between "*interacted*" proteins (positive examples) and "*non-interacted*" proteins (negative examples), the SVM learns a classification function from a set of positive examples *χ*_+ _and set of negative examples *χ*_-_. The classification function takes the form:

(4)

where the non-negative weights *λ*_*i *_are computed during training by maximizing a quadratic objective function and the function *K*(.,.) is called a kernel function [19]. Any new sequence *x *is then predicted to be positive if the function *f*(*x*) is positive. More details about how the weights *λ*_*i *_are computed and the theory of SVM can be found in [31-33].

## Availability and requirements

The datasets can be downloaded from .

## Authors' contributions

NZ has contributed to the conceptual development of PPI-PS, designed and implemented the method, performed the experimental work and the statistical analysis, drafted the manuscript. SL formulated the problem solving algorithm. WE contributed to the implementation of PPI-PS. PC contributed to the manuscript writing. All authors read and approved the final manuscript.
